# Quantitative microbiome profiling of honey bee (*Apis mellifera*) guts is predictive of winter colony loss in northern Virginia (USA)

**DOI:** 10.1038/s41598-024-61199-9

**Published:** 2024-05-14

**Authors:** David B. Carlini, Sundre K. Winslow, Katja Cloppenborg-Schmidt, John F. Baines

**Affiliations:** 1 Department of Biology, American University, 4400 Massachusetts Ave. NW, Washington, DC 20016 USA; 2 Section of Evolutionary Medicine, Institute for Experimental Medicine, Kiel University, Arnold-Heller-Str. 3, 24105 Kiel, Germany; 3 Max Planck Institute for Evolutionary Biology, August-Thienemann-Str. 2, 24306 Plön, Germany

**Keywords:** Ecology, Microbiology, Zoology

## Abstract

For the past 15 years, the proportion of honey bee hives that fail to survive winter has averaged ~ 30% in the United States. Winter hive loss has significant negative impacts on agriculture, the economy, and ecosystems. Compared to other factors, the role of honey bee gut microbial communities in driving winter hive loss has received little attention. We investigate the relationship between winter survival and honey bee gut microbiome composition of 168 honey bees from 23 hives, nine of which failed to survive through winter 2022. We found that there was a substantial difference in the abundance and community composition of honey bee gut microbiomes based on hive condition, *i.e*., winter survival or failure. The overall microbial abundance, as assessed using Quantitative Microbiome Profiling (QMP), was significantly greater in hives that survived winter 2022 than in those that failed, and the average overall abundance of each of ten bacterial genera was also greater in surviving hives. There were no significant differences in alpha diversity based on hive condition, but there was a highly significant difference in beta diversity. The bacterial genera *Commensalibacter* and *Snodgrassella* were positively associated with winter hive survival. Logistic regression and random forest machine learning models on pooled ASV counts for the genus data were highly predictive of winter outcome, although model performance decreased when samples from the location with no hive failures were excluded from analysis. As a whole, our results show that the abundance and community composition of honey bee gut microbiota is associated with winter hive loss, and can potentially be used as a diagnostic tool in evaluating hive health prior to the onset of winter. Future work on the functional characterization of the honey bee gut microbiome’s role in winter survival is warranted.

## Introduction

The honey bee is one of the most important species in domestic agriculture: one-third of the food eaten in the United States is derived from honey bee pollinated crops^1^. In addition to agricultural crops, a large number of ecologically important plant species are pollinated by honey bees^2^. Since 2006–2007, the proportion of honey bee hives that do not survive through the winter months has averaged roughly 30% in the United States^3^. Winter colony loss can be attributed to many factors, but when the worker bees suddenly depart from the hive and leave behind a queen, some nurse bees, immature bees, brood, and ample food, the phenomenon is termed Colony Collapse Disorder (CCD). CCD first appeared in the United States and Europe during the winter of 2006–2007 and was responsible for roughly 50% of hive loss that year. Fortunately, rates of CCD have decreased somewhat over the past five years, accounting for 15–25% of all winter hive loss^4^.

Regardless of the reason, hive loss is a serious threat to agriculture and ecosystem function, and any research that increases our understanding of contributing factors will help in developing approaches to reduce its frequency of occurrence. Winter hive loss has been attributed to abiotic factors, biotic factors, nutrition, beekeeping practices, or some combination thereof. Abiotic factors include pesticide use^5^ as well as temperature and precipitation^6^. Biotic factors include viral^7^, bacterial^8^, or fungal pathogens^9^, mite infestation^10^, or hive invasions by small hive beetles^11^, wax moths^12^, or wasps^13^. Relative to mite loads and pathogens, one biotic factor that has received comparatively little attention is the composition of honey bee gut microbiomes.

The first large scale study to exam microbiome composition in relation to hive loss did not find any clear-cut differences in microbial abundance related to hive loss^14^. However, this early study revealed that, across geographically diverse populations, the honey bee gut microbiome composition is simple, consisting of a core group of less than a dozen taxa. Subsequent studies characterized the microbiota of the honey bee gut community in more detail and established honey bees as a model for gut microbiome research^15–18^. The eusocial behavior of honey bees is an important determinant of their gut microbiome since the microbiota are transmitted vertically between the generations rather than by seeding from the environment^15^. While a core group of microbial taxa are common to honey bees from diverse environments, the proportional composition of each taxon can vary from one colony to the next^19^, and differences in microbial composition might impact colony health through a variety of mechanisms (endocrine signaling and behavior, metabolism, and immune function). Antibiotic-induced alteration of honey bee gut microbiota negatively impacted survival and increased susceptibility to opportunistic pathogens and *Nosema ceranae*^20,21^. Thus, given that experimentally induced changes in the composition of the honey bee gut microbiome can negatively impact hive health, additional research is required to determine the extent to which natural variation in gut microbiomes contributes to colony loss. Toward that end, here we investigate the relationship between winter hive loss, the most common time for hives to fail^22^, and honey bee gut microbiome composition.

## Methods

### Hive sampling

Adult forager bees were collected in July 2021 from 23 hives in three geographically proximate locations in northern Virginia, twelve hives from Gainesville, VA (38.82° N, 77.60° W), nine hives from Upperville, VA (38.97° N, 77.85° W), and two hives from Vienna, VA (38.92° N, 77.25° W) (Table S1). Between 5 and 8 foragers (average 7.3 bees per hive) were collected from the front entrance of each hive, immediately placed in conical tubes containing 50 mL of 100% ethanol (one 50 mL conical tube per hive), transported back to laboratory, and stored at 4 °C until further processing. Hive condition was classified as “survived” or “failed” according to the status of the hive on March 31, 2022, with all hive failures having occurred during the period between December 2021 and March 2022. Eight of the twelve hives sampled from the Gainesville location failed, none of the nine hives sampled from the Upperville location failed, and one of the two hives sampled from the Vienna location failed. All hives had been treated for mites in the spring and fall prior to sampling, and all hives had received supplementary feeding of sugar syrup during the late autumn and winter months; none of the 23 hives had ever been treated with antibiotics, and no visible pathogens such as chalkbrood, foulbrood, or symptoms of *Nosema* infection were detected.

### DNA extraction

Prior to dissection of the gastrointestinal tract, each bee was rinsed with 100% ethanol and then placed in its own sterile disposable petri dish for dissection. The entire intestinal tract was removed from each bee using sterile dissection tools and placed in an individual microcentrifuge tube containing 100% ethanol, and the remaining carcass of each bee, to be used for RNA extractions, was placed in a separate microcentrifuge tube containing 100% ethanol. Prior to DNA extraction, the ethanol was removed, and the intestinal tract was washed twice in sterile PBS. The PBS wash was then removed, and 100 μL of sterile PBS added prior to homogenization with a sterile micropestle. Following homogenization, an additional 900 μL of sterile PBS was added and the tubes were gently mixed and centrifuged at 10,000 × *g* for one minute. Microbial genomic DNA was extracted from the supernatant using the DNeasy® UltraClean® Microbial Kit (Qiagen) following the manufacter’s protocol using a final elution volume of 50 μL EB buffer. DNA was quantified with a NanoVue™ Plus spectrophotometer (GE Healthcare), and stored at − 80 °C.

### 16S rRNA gene sequencing and sequence processing

The hypervariable V1-V2 region of the 16S rRNA gene from each sample was amplified using 27F and 338R primers barcoded with unique octameric multiplex identifiers using a dual indexing approach on an Illumina MiSeq sequencing platform, as previously described^23^. Eight negative extraction controls and three ZymoBIOMICS Microbial Community DNA Standards (Zymo Research) were also sequenced. DADA2 v 1.22^24^ was employed to quality filter sequencing reads, infer amplicon sequence variants (ASVs), and determine taxonomic classification based on the Silva Project v138.1 reference database^25^. The ratio of microbe DNA to honey bee host DNA was determined via quantitative PCR using eubacterial 16S rRNA primers Eub338F and Eub518R^26^ and honey bee acetylcholinesterase 2 primers^27^ Total bacterial load estimates, obtained from the qPCR data, along with the negative extraction controls, were used as input to identify and remove contaminants using the decontam R software package (v1.10.0)^28^ using the “prevalence” method with a threshold of 0.05. Rare ASVs not detected in more than 3 reads in at least 20% of the samples were also excluded from analysis, resulting in 77 retained ASVs. The 16S rRNA gene copy number for each ASV was obtained based on genus assignment using the Ribosomal RNA Database^29^. Sixty-four of the 77 ASVs had a 16S rRNA gene copy number of four. Of the remaining 13 ASVs, ten had a 16S rRNA copy number of two, one had a copy number of three, and two had a copy number of five. ASV counts were weighted by copy number (i.e., counts from high copy number ASVs were reduced, and counts from low copy number ASVs were increased), and the values scaled so that the sum of the scaled counts for each bee sample were the same after copy number correction.

### Quantitative microbiome profiling and microbial community analysis

The qPCR-based bacterial load estimates were used to determine the relative abundance of each sample for quantitative microbiome profiling (QMP)^30^. The sampling depth of reads from each bee gut was calculated as the total number sequencing reads divided by the sample relative abundance. Pooled reads from each bee gut were then rarefied to the sampling depth of the bee gut with minimal sampling depth (bee gut 21-116S-2, sampling depth = 25,348), which rarefied all to an even sampling depth, the ratio between sequencing depth and bacterial load^30^. Sampling depth rarefaction was repeated 1000 times, and the average of the 1000 replicates was used for relative total abundance of each ASV for each bee gut.

Microbial community analysis was conducted in R (v4.2.3)^31^ using the following software packages. Phyloseq (v.1.42.0)^32^ was used to conduct sampling depth rarefaction, and to calculate pairwise distances. DECIPHER (v2.0)^33^ was used to align sequences and Phangorn (v2.11.1)^34^ employed to calculate distances and construct neighbor-joining phylogenetic trees for use in Phyloseq. The Shannon (*H*), Simpson (*D*), and Pielou’s Evenness (*J*) alpha diversity metrics were calculated with the software package vegan (v2.6.4)^35^, which was also used to conduct Permutational Multivariate Analysis of Variance (PERMANOVA) tests on beta diversity, as well as to perform ordination using non-metric multidimensional scaling (NMDS). The software packages DESeq2 (v.1.38.3)^36^ and indicspecies (v1.7.13)^37^ were used to conduct differential abundance analyses on ASVs. Two-way ANOVAs were performed in base R. Two-way ANOVAs were performed using both hive condition and hive location as categorical factors, allowing us to determine how both factors in combination relate to microbial abundance and diversity, and also whether there is any interaction between those factors. For all tests involving multiple comparisons, *P* values were corrected using a Benjamini and Hochberg adjustment for a type I error rate of 0.05^38^. MikropML (v1.6.0)^39^ was employed to conduct supervised machine learning for classification of the hive condition of individual honey bee samples using L2 logistic regression and random forest algorithms based on the ASVs annotated to the genus level, as fine resolution ASV level analysis has been found to be too individualized for accurate classification^40^.

### RNA extraction and deformed wing virus quantitative PCR

Deformed wing virus (DWV), vectored by the ectoparasitic mite *Varroa destructor*, has been identified as a major biological causative agent of colony loss, and the most regularly detected virus in western honey bees^41,42^. We quantified viral loads of the three main DWV strains via qPCR. Total RNA was extracted from the same bees from which the gut microbiome 16S rRNA gene sequence data was obtained. RNA was extracted using the RNeasy® Plus Universal Mini Kit (Qiagen) and stored at − 80 °C. cDNA was synthesized from extracted RNA using the oligo(dT) primer supplied with the AffinityScript qPCR cDNA Synthesis Kit (Agilent), and qPCR was performed using the Brilliant III Ultra-Fast SYBR Green qPCR Master Mix (Agilent) on the Mx3005P qPCR System (Agilent). To characterize deformed wing virus (DWV) viral loads we used the DWV strain specific A, B, and C primer sequences developed by Kevill et al.^42^ along with GAPDH primers as the control gene^43^. All four genes were assayed in triplicate on the same qPCR run for each sample, along with triplicate negative controls (with water as template for cDNA synthesis) for each gene. The relative abundances of DWV-A, DWV-B, and DWV-C were calculated as DC_T_ (C_T *GAPDH*_−C_T DWV_), the difference between the GAPDH threshold cycle and that of the DWV-A, DWV-B, or DWV-C threshold cycle. Two-way nested ANOVAs, with hive condition and location as factors, were used to assess the statistical significance of DC_T_ values for each DWV strain.

## Results

### Gut microbial composition and abundance

**Figure 1:**
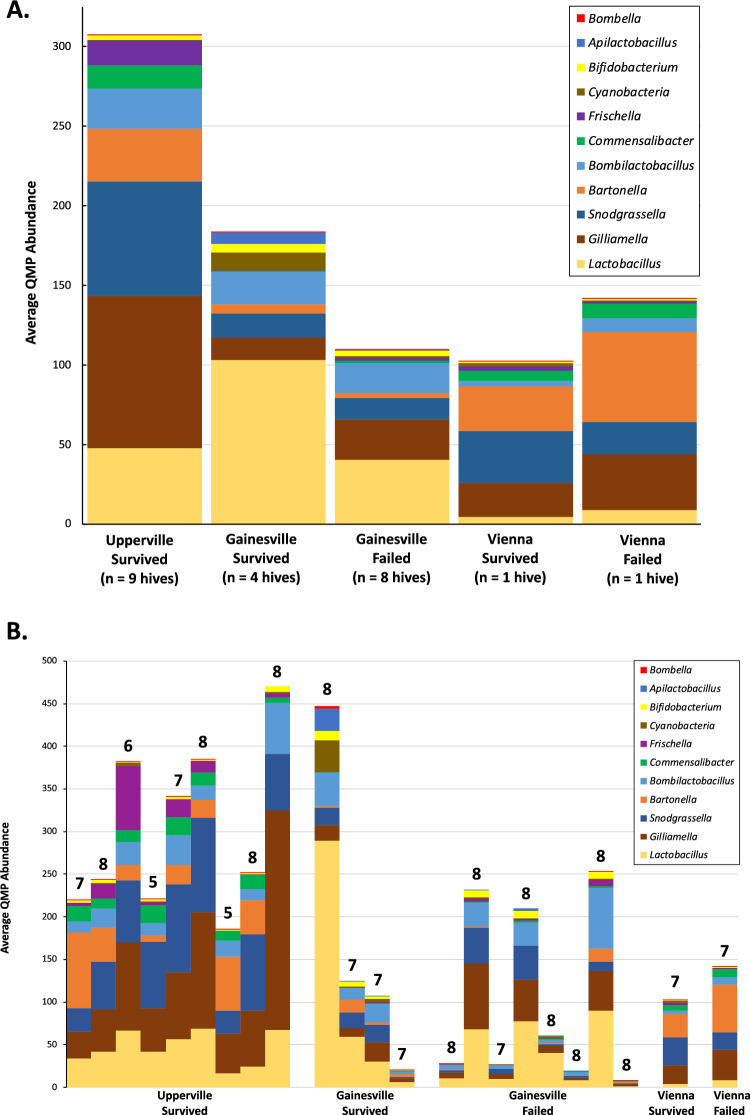
Community composition of honey bee gastrointestinal tracts deduced from quantitative microbiome profiling (QMP). Relative bacterial loads for QMP were obtained via qPCR, with the sampling depth of reads from each bee gut calculated as the total number sequencing reads divided by the sample relative abundance. Pooled reads from each bee gut were then rarefied to the sampling depth of the bee gut with minimal sampling depth, which rarefied all to an even sampling depth, the ratio between sequencing depth and bacterial load. (**A**) Average overall QMP sampling abundance of bacterial genera in hives that survived or failed in each sampling location. (**B**) Average QMP sampling abundance of bacterial genera in each of the 23 sampled hives. The number of bees sampled per hive is given above each stacked bar (5–8 bees per hive, average = 7.3).

The microbial gut communities of 168 bees from 23 hives were sequenced, yielding an average of 36,178 reads (range: 9461–73,402) and 62 ASVs (range: 13–333) per sample after error correction, quality filtering, and removal of potential contaminants. After removal of rare ASVs, a total of 77 ASVs were retained, with an average of 28,098 reads (range: 4148–63,441) and 35 ASVs (range: 10–52) per sample. After adjusting for differences due to different 16S rRNA gene copy number among different taxa and rarefying samples to an even sampling depth (the ratio between sequencing depth and bacterial load), overall QMP abundance was significantly greater in samples from hives that survived winter 2022 than in those that failed (*P* = 0.0136, nested two-way ANOVA), whereas hive location and hive condition × location interaction effects were not statistically significant. Each of the nine hives in the Upperville location survived Winter 2022, and the average QMP sampling abundance of those hives (307 ± 33) was over twofold greater than the average QMP abundance of the eight hives that failed in the Gainesville location (109 ± 38), which was also less than the average of the four hives that survived in the Gaineville location (184 ± 94) (Fig. 1A). The average QMP abundance of the single failed hive in the Vienna location (142 ± 31) was less than the average of the surviving hives from both other locations, but greater than that of the single surviving hive from the Vienna location (103 ± 25). However, there was substantial variation in QMP abundance among hives within each sampling location and hive condition category, particularly at the Gainesville location (Fig. 1B).

**Figure 2:**
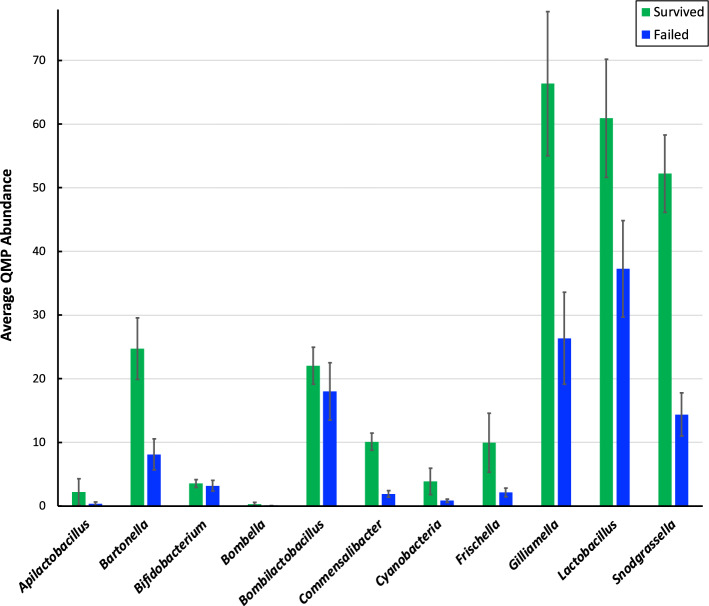
Pairwise comparisons of average QMP sampling abundance of ten most abundance bacterial genera of hives that survived (green) or failed to survive (blue) winter 2022. Error bars indicate standard error of the mean.

The honey bee gut microbiome is dominated by five core genera, *Bifidobacterium*, *Bombilactobacillus*, *Gilliamella*, *Lactobacillus*, and *Snodgrassella*, which have high abundance and prevalence, and four to five noncore genera which are typically found at lower frequencies and prevalence than the core genera^19^. Each of the five core genera had high prevalence, being present in at least 98% of the 168 individual honey bee samples. The noncore genera *Apilactobacillus*, *Bartonella*, *Bombella*, *Commensalibacter*, and *Frischella* had prevalences in individual bees of 58, 71, 42, 89, and 68%, respectively. For each of these ten bacterial genera, the average quantitative sampling abundance was greater in honey bees from surviving hives than in those from failed hives (Fig. 2). In addition to these ten bacterial genera, most hives contained 16rRNA gene hits to “Cyanobacteria”, sequences that are likely derived from the chloroplasts of consumed pollen^15,44^. Sequences of *Paenibacillus larvae* and *Streptococcus pluton*, the causative agents of American and European foulbrood (diseases of bee larvae), were not detected among the 77 ASVs or among the inclusive set of 1465 ASVs recovered prior to filtering.

### Microbial diversity

Within subject species diversity (alpha diversity) was not significantly different between hives that survived and those that failed for Shannon Diversity (*P* = 0.24, two-way ANOVA), Simpson Diversity (*P* = 0.53) or Pielou’s Evennness (*P* = 0.15) (Table S2). Alpha diversity based on hive location was not significant for Shannon or Simpson Diversity (*P* = 0.065, 0.17, respectively), but was significant for Pielou’s Evennness (*P* = 0.015). However, when samples from the Upperville location (where no winter hive loss occurred) were excluded from analysis of alpha diversity, the differences based on hive condition or hive location were not statistically significant for any of the three measures of alpha diversity (Table S2).

**Figure 3:**
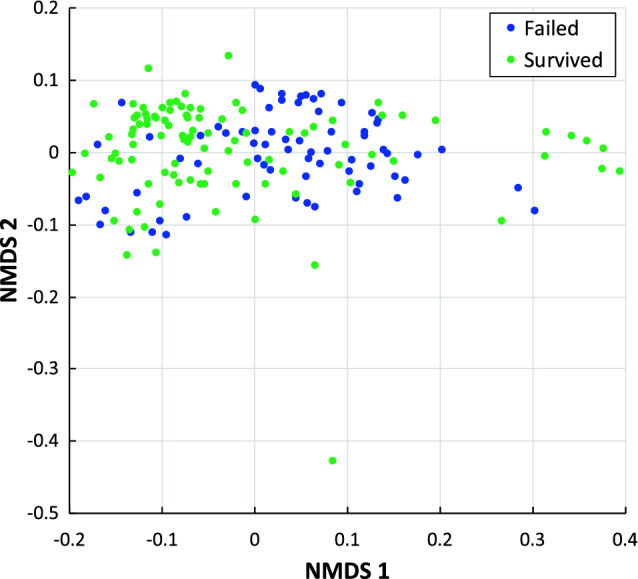
NMDS ordination of honey bee gut microbiota from hives that survived (green) or hives that failed to survive (blue) winter 2022. Weighted UniFrac dissimilarity was calculated using the absolute abundance of 77 ASVs. PERMANOVA performed on the weighted UniFrac distances showed significant effect of hive condition on beta-diversity (*P* < 10^–6^, stress = 0.122).

Next, we visualized the difference in bacterial species composition (beta diversity) by NMDS ordination (Fig. 3) based on the weighted UniFrac distance (ordination stress = 0.122). To compare community composition among hives, we conducted permutational multivariate analysis of variance (PERMANOVA) implemented with the *adonis* method with 10^6^ permutations in the R vegan package (v.2.6.4)^35^. For all three distance metrics used (weighted UniFrac, Bray Curtis, and Jaccard), the effects of hive condition, as well as hive location, were highly significant (PERMANOVA test: *P* < 10^–6^), indicating that microbial community composition differed based on both hive condition and hive location (Table 1). Permutation tests for the assumption of homogeneity of dispersions^45^ confirmed that the assumption was met for all three distance metrics (PERMDISP, adjusted *P* > 0.05). Since each of the hives in the Upperville location survived, it could be argued that most of that signal was due to hive location. We therefore conducted the PERMANOVA analysis after excluding the Upperville samples, but found that hive condition remained significant (P < 10^–4^, Table 2; Supplementary Fig. S1), indicating that the differences in bacterial community composition can be attributed to hive condition.

**Table 1:** Summary of PERMANOVA tests of beta-diversity dissimilarity based on hive condition and location.

Weighted UniFrac distances			
Factor	R2	Pseudo-*F*	*P*
Condition	0.15	37.03	< 10^–6^
Location	0.17	21.13	< 10^–6^
Condition × Location	0.012	2.96	0.036

Bray–Curtis distances			
Factor	R2	Pseudo-*F*	*P*
Condition	0.047	8.98	< 10^–6^
Location	0.089	8.54	< 10^–6^
Condition × Location	0.0085	1.61	0.034

Jaccard distances			
Factor	R2	Pseudo-*F*	*P*
Condition	0.031	5.69	< 10^–6^
Location	0.062	5.60	< 10^–6^
Condition × Location	0.0080	1.44	0.022

**Table 2:** Summary of PERMANOVA tests of beta-diversity dissimilarity based on hive condition and location (Upperville excluded).

Weighted UniFrac distances			
Factor	R2	Pseudo-*F*	*P*
Condition	0.062	9.74	3.40 × 10^–5^
Location	0.26	41.16	< 10^–6^
Condition × Location	0.34	5.35	0.022

Bray–Curtis distances			
Factor	R2	Pseudo-*F*	*P*
Condition	0.015	1.68	0.024
Location	0.073	8.23	< 10^–6^
Condition × Location	0.012	1.32	0.12

Jaccard distances			
Factor	R2	Pseudo-*F*	*P*
Condition	0.013	1.41	0.0249
Location	0.051	5.59	< 10^–6^
Condition × Location	0.012	1.29	0.059

### Differential abundance

Differential abundance analysis was conducted using DESeq2 and indicspecies on QMP ASV counts from all locations, as well as by performing nested two-way ANOVAs on genus QMP abundance. DESeq2 identified 12 ASVs that were associated with hive condition, seven of which were positively associated and five of which were negatively associated with winter survival (Table 3). Indicspecies identified nine ASVs that were positively associated with hive survival (Table 4), six of which were also identified by DESeq2: ASV1 (*Snodgrassella alvi*), ASV2 (*Lactobacillus apis*), ASV3 (*Giliamella apicola*), ASV9 (*Commensalibacter melissae*), ASV13 (*Bartonella apis*), and ASV25 (*Giliamella*).

**Table 3:** ASVs significantly associated with hive condition from DESeq2 analysis.

OTU	Genus	Survived:Failed ratio	Adjusted *P*
ASV1	*Snodgrassella*	4.32	1.67 × 10^–8^
ASV13	*Bartonella*	9.85	6.81 × 10^–8^
ASV25	*Gilliamella*	7.41	2.42 × 10^–7^
ASV3	*Gilliamella*	4.26	1.99 × 10^–6^
ASV2	*Gilliamella*	2.50	5.05 × 10^–6^
ASV16	*Lactobacillus*	0.37	7.94 × 10^–6^
ASV9	*Commensalibacter*	4.11	1.43 × 10^–5^
ASV57	*Lactobacillus*	0.38	6.74 × 10^–4^
ASV65	*Lactobacillus*	0.30	6.74 × 10^–4^
ASV76	*Lactobacillus*	0.24	1.04 × 10^–3^
ASV15	*Snodgrassella*	6.77	1.32 × 10^–3^
ASV73	*Lactobacillus*	0.28	1.32 × 10^–3^

**Table 4:** ASVs significantly associated with hive condition from indicspecies analysis.

OTU	Genus	Adjusted *P*
ASV1	*Snodgrassella*	0.0015
ASV2	*Gilliamella*	0.0015
ASV3	*Gilliamella*	0.0015
ASV9	*Commensalibacter*	0.0015
ASV25	*Gilliamella*	0.0015
ASV51	*Bombilactobacillus*	0.013
ASV35	*Bartonella*	0.032
ASV13	*Bartonella*	0.043
ASV44	*Gilliamella*	0.048

Nested two-way ANOVAs on pooled QMP ASV counts for each genus identified that two genera were positively associated with winter survival while accounting for location, *Commensalibacter* and *Snodgrassella* (Table 5).

**Table 5:** Nested two-way ANOVA tests on genus abundance from pooled ASV counts.

Genus	Condition (Adjusted *P*)	Location (Adjusted *P*)	Condition × Location (*P*)
*Commensalibacter*	9.3 × 10^–5^	1.3 × 10^–5^	0.68
*Snodgrassella*	4.5 × 10^–3^	0.01	0.76
*Bartonella*	0.16	0.033	0.30
*Gilliamella*	0.21	0.11	0.98
*Frischella*	0.44	0.30	0.89
*Lactobacillus*	0.53	0.36	0.49
*Apilactobacillus*	0.55	0.34	0.43
*Bombella*	0.60	0.27	0.47
*Bombilactobacillus*	0.71	0.41	0.83
*Bifidobacterium*	0.82	0.54	0.71

### Quantitative PCR assays on DWV levels

There were no significant differences in DWV loads based on hive condition (Table S3). For Gainesville and Vienna (the two locations containing both hives that survived and failed winter 2022), hives that failed winter 2022 exhibited higher average DWV-A, DWV-B, and DWV-C loads than hives that survived winter 2022, although these differences were not statistically significant. There were also no significant differences in DWV-A and DWV-B loads based on hive location. DWV-C loads were significantly different among locations (*P* = 0.0087, two-way ANOVA), due to lower loads of DWV-C in the Gainesville location (average DC_T_ = − 0.94) compared to the Upperville (average DC_T_ = 0.55) and Vienna (average DC_T_ = 0.42) locations.

### Machine learning classification of winter hive loss

**Figure 4:**
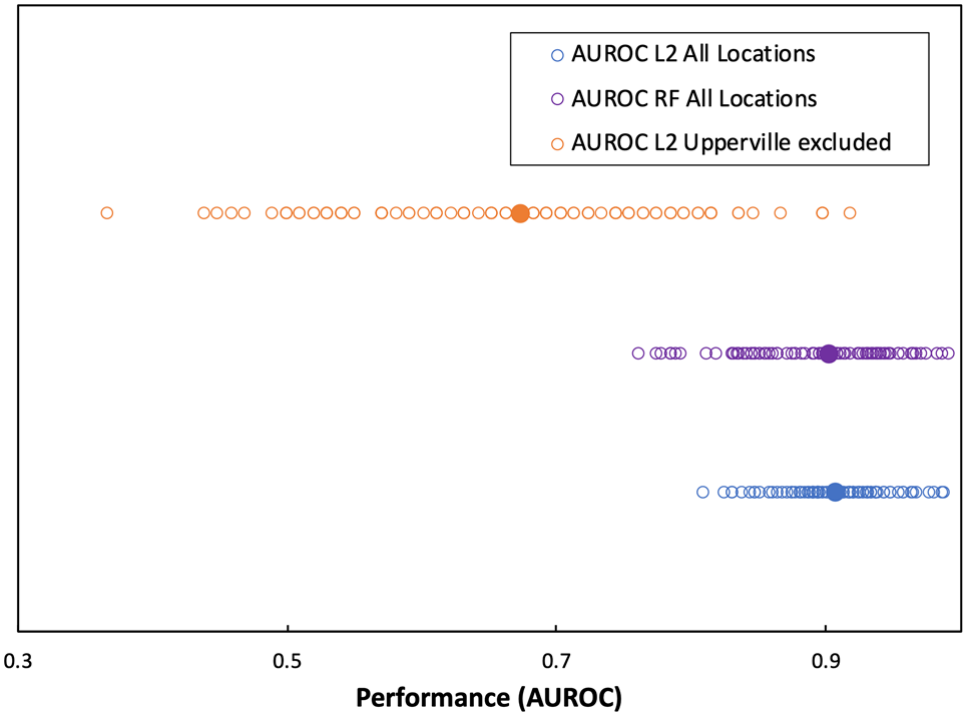
Model performance of machine learning methods, as measured by the area under the receiver operator characteristic curve (AUROC). Strip plots of AUROC values on the test data set for each of the 100 seeds using a L2 logistic regression model (blue), random forest model (purple), or L2 logistic regression model with the Upperville samples excluded (orange), with the median AUROC values for each depicted as filled circles. An AUROC value of 0.5 corresponds to random classification, whereas an AUROC value of 0.9, the approximate median of the 100 seeds for both the L2 logistic regression and random forest ML models with all locations excluded, corresponds to a classification system that correctly identifies a sample as coming from a surviving or failed hive with 90% probability.

To gain a better understanding of the relative importance of each factor contributing to hive loss, we employed supervised machine learning, as implemented in the R software package MikropML^39^, using two machine learning (ML) models, L2 logistic regression and random forest. We performed a grid search for hyperparameter settings when training the L2 logistic regression models. For each model, we ran 100 iterations, with a split of 80% of the data for the training and 20% for testing. The performance of the two methods, as measured by the area under the receiver operator characteristic curve (AUROC), illustrate the predictive power of a binary classifier, in this case whether a particular sample comes from a hive which failed or survived winter 2022 (Fig. 4). The overall performance of the two methods was similar, where the median AUROC was 0.908 and 0.903 for the L2 logistic regression and random forest models, respectively. For comparison, we also employed a L2 logistic regression model excluding the samples from the Upperville location, where no hive failures occurred, which resulted in a substantial drop in performance (median AUROC = 0.673).

**Figure 5:**
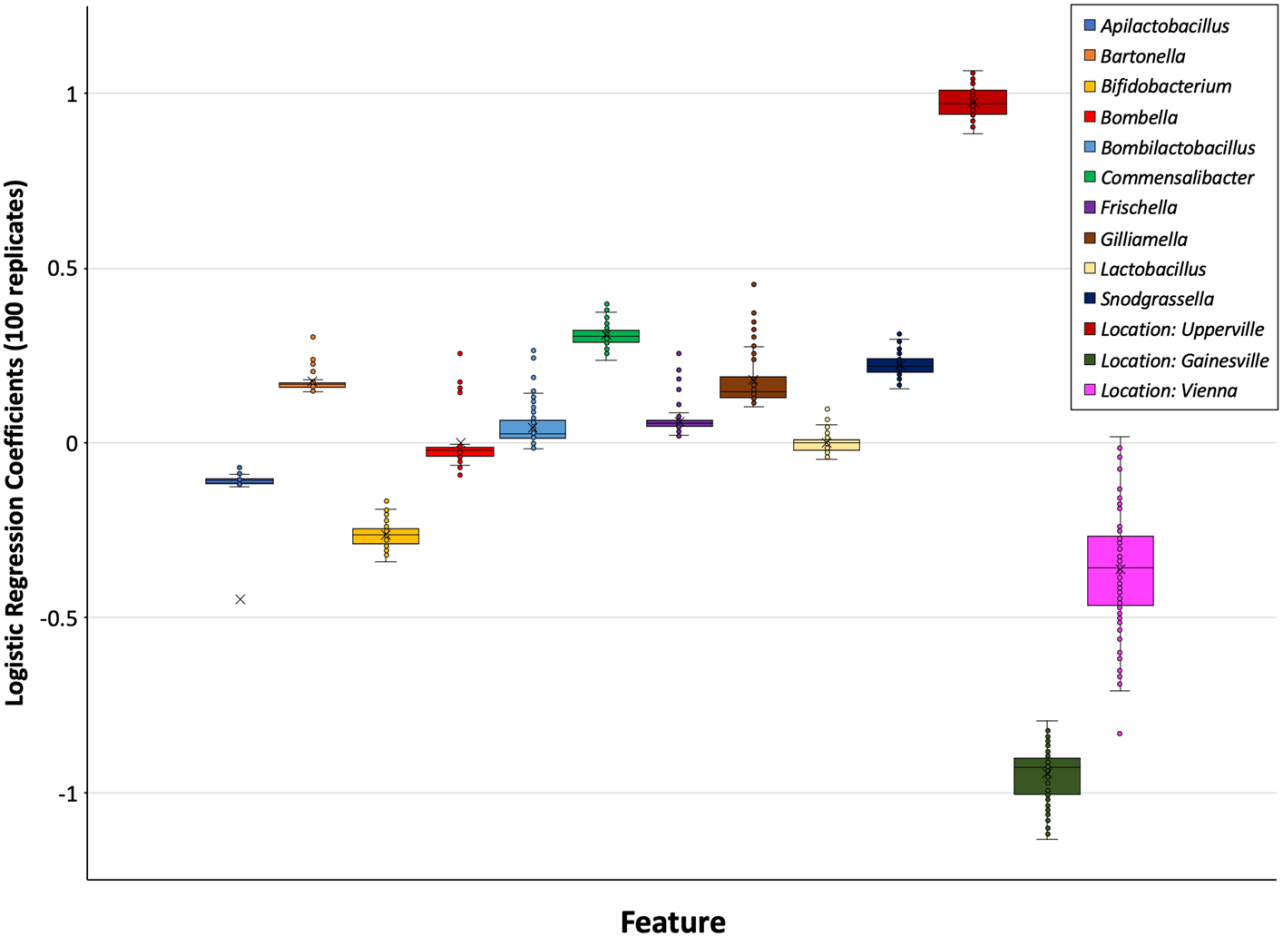
Boxplot of L2 regression model feature coefficients (*i.e*., weights) for each feature. Features associated with winter survival have positive regression coefficients (*e.g*., genera *Commensalibacter* and *Snodgressella*, location Upperville), and those associated with winter failure have negative regression coefficients (e.g. genus *Bfidobacterium*, locations Gainesville and Vienna), with the magnitude proportional to feature importance in the ML model. The medians and interquartile ranges of the 100 seeds are depicted as horizontal black lines and boxes, respectively.

For L2 logistic regression, an intuitive way to evaluate the relative contributions of each feature (*i.e*., genus or location) to model performance is to compare the regression coefficients (*i.e*., weights) for each feature, where the magnitude is proportional to feature importance in the ML model. As expected, given that each of the hives from the Upperville location survived and that two-thirds of the hives from the Gainesville location failed, the average regression coefficients of these two location features were of the greatest magnitude, 0.97 and − 0.93, respectively (Fig. 5). Of the 10 taxonomic features, four were positively associated (average regression coefficient > 0.1) with hive survival (*Bartonella*, *Commensalibacter*, *Gilliamella*, *Snodgrassella*), two were negatively associated (average regression coefficient < − 0.1) with hive survival (*Apilactobacillus*, *Bfidobacterium*), and four were weakly associated (− 0.1 < average regression coefficient < 0.1) with hive survival (*Bombella*, *Bombilactobacillus*, *Frischella*, *Lactobacillus*). Another means of determining the relative importance of each feature is through permutation, where model performance is compared between the inclusive data and when each feature is omitted. As expected, exclusion of the Upperville and Gainesville location features had the greatest impact on model performance (~ 8.5–10% drop in median AUROC), followed by the taxonomic features *Bfidobacterium*, *Commensalibacter*, *Snodgrassella*, and *Gilliamella* (~ 2–5% drop in median AUROC), with the remaining taxonomic features having a lesser to negligible effect on model performance (< 2% drop in median AUROC).

## Discussion

In this study, we compared the gut microbiota in honey bees from hives in three sampling locations that either survived or failed to survive through winter 2022. The bacterial taxa found in this study included the six Gram-negative genera *Bartonella*, *Bombella* (formerly *Parasaccharibacter*), *Commensalibacter*, *Frischella*, *Gilliamella*, and *Snodgrassella*, as well as the four Gram-positive genera *Apilactobacillus* (formerly *Lactobacillus*), *Bifidobacterium*, *Bombilactobacillus* (formerly *Lactobacillus*), and *Lactobacillus*. These taxa are consistent with previous work demonstrating that the honey bee gut microbiome is simple and consists of less than a dozen species clusters^16,17,19,46^.

The most salient difference between the bacteriomes of honey bees from hives that survived versus those that failed is in total bacterial abundance, where bacterial abundance of hives that survived consistently exceeded that of hives that failed. Importantly, for *each* of the ten bacterial genera the average QMP abundance was greater in honey bees from surviving hives (Fig. 2). Given that all 10 genera are well known commensals of honey bees, perhaps an overall dearth of gut commensals, which are critical for metabolism, endocrine signaling/growth, immune function, and pathogen resistance^47^, led to various forms of stress (e.g., lack of nutrition, increased susceptibility to pathogens), contributing to winter hive failure. Consistent with the hypothesis that greater abundance of members of the core bee gut microbiome is protective, a survey of the gut microbiota of thriving versus non-thriving honey bees found higher relative abundances of *Bartonella*, *Bifidobacterium*, *Bombella*, *Commensalibacter*, and *Snodgrassella* in thriving bees^48^. In another recent study, winter bees, which are crucial for colony survival, were found to have roughly tenfold higher total gut bacterial loads than summer foragers^49^, which suggests a relationship between the health of winter hives and total bacterial abundance under a scenario in which hives with particularly low total bacterial abundance in their summer foragers are more likely to exhibit low bacterial abundance in the winter.

Whereas the difference wasn’t statistically significant for two-way ANOVAs performed on most genera, the pooled ASVs from two genera, *Commensalibacter* and *Snodgrassella*, exhibited significantly greater abundances based on hive condition. The average abundance of *Commensalibacter*, a member of the acetic acid bacteria (family Acetobacteraceae), among honey bees from hives that survived was, on average, over five-fold higher than that from those from hives that failed. In addition, the average regression coefficient for *Commensalibacter* as a feature in a logistic regression ML model was of greater magnitude (positive or negative) than that of any other genus, indicating that a greater abundance of the *Commensalibacter* was more predictive of hive success than any other gut commensal. In a comparative study of thriving versus non-thriving honey bees, *Commensalibacter* was found to be significantly more abundant in the gut of thriving bees compared to non-thriving bees^48^, and *Commensalibacter* abundance has been found to increase in winter bees relative to summer foragers^49^. In *Drosophila*, *Commensalibacter* suppresses the proliferation of a pathogenic commensal *Gluconobacter morbifer*^50^.

The average abundance of *Snodgrassella* among honey bees from hives that survived was, on average, almost four-fold higher than that from honey bees from failed hives and, similar to *Commensalibacter*, *Snodgrassella* abundance was predictive of hive success in a logistic regression ML model. *Snodgressella alvi* is a member of the core gut microbiome, it has been found in almost every adult honey bee worker worldwide, and is most abundant in the ileum region of the hindgut^19^. *S. alvi* has been found to protect against *E. coli* hemolymph infection^51^, and is known to play a very important role in maintaining anoxia in the gut, a condition required by the metabolism of other gut symbionts^52^. After infection with *E. coli*, honey bees mono-inoculated with *S. alvi* cleared more *E. coli* from the hemolymph after infection, and they had higher levels of antimicrobial peptide, so it has been proposed that *S. alvi* may have a role in immune priming^49^. Consistent with this finding, recent studies determined that *S. alvi*, which forms a dense biofilm in the ileum, triggered an immune response against the opportunistic and harmful microbial pathogen *Serratia marescens*, protecting them against infection^53,54^. *Serratia marescens* infection is unlikely to be a factor influencing hive survival in our study given that *Serratia* was only detectable in three samples prior to filtering out rare ASVs. *Serratia* hits accounted for < 1% of the total reads from each of the three individual honey bee samples in which it was detected, and two of the three samples were from two different hives that survived winter 2022. *S. alvi* has also been shown to protect against *Paenibacillus larvae*^55^, the microbial pathogen responsible for American foulbrood, the most widespread disease affecting honey bee larvae. We did not detect any reads from American foulbrood prior to filtering out rare ASVs, therefore, similar to *Serratia*, any protection *S. alvi* conferred against *P. larvae* was probably not a factor influencing hive survival in our study. However, *S. alvi* may protect against a variety of pathogens, not all of which would be detectable using bacterial 16SrRNA gene sequencing. A seasonal shift in the abundance of *S. alvi* occurs in the midgut, where it becomes more abundant in summer, possibly due to changes in diet^56^. It is possible that lower levels of *S. alvi* are indicative of poor nutrition, resulting in higher disease susceptibility and mortality.

Contrary to results from a previous study^48^ that found higher alpha diversity in the gut microbiomes of thriving hives (rapid hive population growth, high honey production) compared to non-thriving hives (slow hive population growth, low honey production), we found no difference in the alpha diversity of hives that survived or failed winter 2022. Whether increased microbial diversity is a signature of hive health depends on which species most contribute to increased diversity, *e.g*., the presence of rarer pathogenic bacteria could increase species richness but may have a detrimental effect on hive health. There was a marginally significant difference in species evenness based on hive location, with the Gainesville location having higher diversity (average Pielou’s evenness = 0.87) than the Upperville and Vienna locations (average Pielou’s evenness = 0.72 and 0.78, respectively). Given that the Gainesville location had the highest rates of hive failure, this is a case where higher gut microbial diversity is not necessarily a sign of hive health (average Shannon and Simpson diversity were highest for the Gainesville location as well, but the differences among locations were not statistically significant).

In contrast to some previous studies on colony loss^41,44,57^, we did not find any evidence that any of the three DWV strains was a significant driver of colony failure. The lack of any significant differences in DWV levels based on hive condition may be attributed to the fact that each of the hives we sampled had been treated during the spring and fall for parasitic *Varroa destructor* mites, an important vector of DWV and other viruses. It is possible that, in the ensuing months between the fall mite treatment and winter, mite loads and the concomitant DWV levels accrued in some hives, perhaps contributing to some hive failures.

In addition to differences in specific taxa, the difference in overall composition of hives that survived versus those that failed was highly significant (*P* < 10^–6^) as determined from a PERMANOVA test of beta diversity differences based on hive condition. Although the effect of hive location was also highly significant (*P* < 10^–6^), the PERMANOVA test remained highly significant (*P* < 10^–4^) even after excluding samples from the Upperville location, where all hives survived winter 2022. These results indicate that there are consistent differences in the abundances of individual taxa in surviving versus failed hives, although the specific nature of these differences is difficult to discern due to the multidimensionality of the beta diversity data.

To aid in the interpretation of PERMANOVA results we used ML models to determine if the microbial community composition of honey bee guts were predictive of winter hive survival or failure, as well as to determine which taxonomic features were most strongly associated with survival or failure. Both the logistic regression and random forest ML models performed very well (median AUROC > 0.9) when all features were included, however, since all samples from the Upperville location were obtained from hives that survived the winter and two-thirds of the hives from the Gainesville location failed, the location features contributed significantly to ML performance. Despite the predictive importance of the Upperville location feature, when the Upperville samples were excluded the logistic regression ML model remained predictive of winter survival (median AUROC = 0.673), indicating that the taxonomic composition of the honey bee gut microbiome alone was an important determinant of winter failure and could be used to correctly predict whether a particular honey bee sample came from a failed hive ~ 67% of the time. In accordance with the results from statistical analysis of individual genus abundance data discussed above, the *Commensalibacter* and *Snodgrassella* genera were the taxonomic features that were most predictive of hive success, having the largest regression coefficients in the logistic regression model, and having the greatest impact on model performance when excluded as features in the logistic regression and random forest ML models.

Given that (1) most colony loss occurs during the winter months, that honey bee gut microbial communities undergo seasonal fluctuations^47^, (2) there are clear differences in the gut microbial communities of thriving versus non-thriving bees during the summer^46^, (3) there is a significant difference between the gut microbial communities of honey bees from hives that were destined for winter failure and those that survived (this study), and (4) that this difference has predictive power (this study), further analysis and a functional characterization of the honey bee gut microbiome’s role in winter survival is clearly warranted.

This study suggests that honey bee gut microbial abundance and community composition may play a significant role in winter hive loss. Honey bees from hives that survived winter 2022 had significantly higher microbial loads, and there was a highly significant difference in the beta diversity based on hive condition. Two bacterial genera previously demonstrated to be beneficial, *Commensalibacter* and *Snodgrassella*, were also found to be positively associated with winter survival in our study. Machine learning models were predictive of hive outcome, indicating that in the future the community composition honey bee gut microbiota can potentially be used as a diagnostic tool in evaluating hive health prior to the onset of winter.

### Supplementary Information

Supplementary Information.

## Data Availability

The 16S rRNA gene reads supporting the conclusions of this article are available in the NIH Sequence Read Archive repository (https://www.ncbi.nlm.nih.gov/sra) under the Bioproject ID PRJNA1010874. R scripts used for microbiome profiling are available upon request.
