# Supplementary material for: Quantitative microbiome profiling of honey bee (Apis mellifera) guts is predictive of winter colony loss in northern Virginia (USA)
Source: Sci Rep. 2024 May 14;14:11021. doi: 10.1038/s41598-024-61199-9 (PMC11094147; doi:10.1038/s41598-024-61199-9)
Supplement: Supplementary file 1 — Supplementary Information. [file 41598_2024_61199_MOESM1_ESM.docx]

**Fig. S1.** NMDS ordination of honey bee gut microbiota from hives that survived (green) or hives that failed to survive (blue) winter 2022 exclusing hives from the Upperville location. Weighted UniFrac dissimilarity was calculated using the absolute abundance of 77 ASVs. PERMANOVA performed on the weighted UniFrac distances showed significant effect of hive condition on beta-diversity (*P* < 10^-4^, stress = 0.125).

**Table S1. Sampling information for honey bee samples analyzed in this study.**

| **Location** | **Hive** | **Sampling Date** | **Sample Size^a^** | **March 31, 2022 Condition** |
| --- | --- | --- | --- | --- |
| Upperville | 21-116S | 7/24/2021 | 6 | Survived |
| Upperville | 21-120 | 7/24/2021 | 5 | Survived |
| Upperville | 21-121 | 7/24/2021 | 7 | Survived |
| Upperville | 21-122 | 7/24/2021 | 8 | Survived |
| Upperville | 21-135 | 7/24/2021 | 5 | Survived |
| Upperville | 21-164 | 7/24/2021 | 8 | Survived |
| Upperville | 21-166 | 7/24/2021 | 8 | Survived |
| Upperville | 20-194 | 7/24/2021 | 7 | Survived |
| Upperville | 20-195 | 7/24/2021 | 8 | Survived |
| Gainesville | 21-112 | 7/17/2021 | 8 | Failed |
| Gainesville | 21-151 | 7/17/2021 | 8 | Failed |
| Gainesville | 21-152 | 7/17/2021 | 7 | Failed |
| Gainesville | 21-153 | 7/17/2021 | 8 | Failed |
| Gainesville | 21-154 | 7/17/2021 | 8 | Failed |
| Gainesville | 21-155 | 7/17/2021 | 7 | Survived |
| Gainesville | 21-156 | 7/17/2021 | 8 | Failed |
| Gainesville | 21-157 | 7/17/2021 | 7 | Survived |
| Gainesville | 21-158 | 7/17/2021 | 8 | Failed |
| Gainesville | 21-159 | 7/17/2021 | 8 | Survived |
| Gainesville | 20-159 | 7/17/2021 | 8 | Failed |
| Gainesville | JSD 21 | 7/17/2021 | 7 | Survived |
| Vienna | Alenka | 7/22/2021 | 7 | Survived |
| Vienna | Ursula II | 7/22/2021 | 7 | Failed |

^a^Number of bee gut microbiomes characterize per hive after filtering out potentially contaminated samples with the decontam R software package (see methods).

**Table S2. *P*-values from two-way ANOVA statistical tests on measures of alpha diversity.**

| **All Locations Included** | | | |
| --- | --- | --- | --- |
|  | **Shannon Diversity (*H*)** | **Simpson Diversity (*D*)** | **Pielou’s Evenness (*J*)** |
| Condition | 0.24 | 0.53 | 0.15 |
| Location | 0.065 | 0.17 | 0.015 |
| Interaction (Condition x Location) | 0.93 | 0.99 | 0.99 |
| **Upperville Location Excluded** | | | |
|  | **Shannon Diversity (*H*)** | **Simpson Diversity (*D*)** | **Pielou’s Evenness (*J*)** |
| Condition | 0.65 | 0.56 | 0.55 |
| Location | 0.45 | 0.67 | 0.33 |
| Interaction (Condition x Location) | 0.94 | 0.99 | 0.99 |

**Table S3. Deformed wing virus loads (average ΔCt) and nested two-way ANOVA results**

| **DWV-A Average ΔCt (=Ct*_GAPDH_* – Ct*_DWV-A_*)** | | | **DWV-A Two-way ANOVA** | | |
| --- | --- | --- | --- | --- | --- |
| **Location** | **Survived** | **Failed** | **Factor** | ***F*** | ***P*** |
| Upperville | –1.39 | N/A^a^ | Condition | 1.054 | 0.47 |
| Gainesville | –3.03 | –1.53 | Location | 2.14 | 0.10 |
| Vienna | –0.69 | –0.57 | Condition x Location | 0.435 | 0.52 |
| **DWV-B Average ΔCt (=Ct*_GAPDH_* – Ct*_DWV-B_*)** | | | **DWV-B Two-way ANOVA** | | |
| **Location** | **Survived** | **Failed** | **Factor** | ***F*** | ***P*** |
| Upperville | –0.33 | N/A | Condition | 0.139 | 0.68 |
| Gainesville | –2.43 | –1.82 | Location | 2.542 | 0.11 |
| Vienna | –0.16 | 0.25 | Condition x Location | 0.005 | 0.92 |
| **DWV-C Average ΔCt (=Ct*_GAPDH_* – Ct*_DWV-C_*)** | | | **DWV-C Two-way ANOVA** | | |
| **Location** | **Survived** | **Failed** | **Factor** | ***F*** | ***P*** |
| Upperville | 0.55 | N/A | Condition | 2.702 | 0.11 |
| Gainesville | –2.37 | –0.12 | Location | 6.786 | 0.009 |
| Vienna | –0.11 | 0.83 | Condition x Location | 0.746 | 0.40 |

^a^There were no Winter 2022 hive failures in the Upperville location.
